# Supplementary material for: Zebrafish Polymerase Theta and human Polymerase Theta: Orthologues with homologous function
Source: PLoS One. 2025 Apr 29;20(4):e0321886. doi: 10.1371/journal.pone.0321886 (PMC12040184; doi:10.1371/journal.pone.0321886)
Supplement: S1 Fig — Red indicates loop insertions, thumb residues blue, fingers residues red, palm residues green, and exo-nuclease residues yellow. (PDF) [file pone.0321886.s001.pdf]

```
hPolθ PD    GFKDNSPISDTSFSLQLSQDGLQLTPASSSSESLSIIDVASDQNLFQTFIKEWRCKKRFS     1851
zPolθ PD    -----------------------------------IIDVASDRRLFETFVNEWKTKERFS     1857
                                               *******:.**:**::**: *:***

hPolθ PD    ISLACEKIRSLTSSKTATIGSRFKQASSPQEIPIRDDGFPIKGCDDTLVVGLAVCWGGRD     1911
zPolθ PD    LAVACEKTDSTSVQPETVIGGKFKKPTTPMR-NKRKDGFLLKGYEDLVVIGISVSWGAKD     1916
            :::****  * : .   :.**.:**: ::* .    *.*** :** :* :*:*::*.**.:*

hPolθ PD    AYYFSLQKEQKHSEISASLVPPSLDPSLTLKDRMWYLQSCLRKESDKECSVVIYDFIQSY     1971
zPolθ PD    AYFVSLQQELVDTDISASLAPPPLDDTLTVEERLKQIQSCLQKDSS---VTVTYDFIHLY     1973
            **:.***:*  .::*****.** ** :**:::*:  :****:*:*.     .* ****: *

hPolθ PD    KILLLSCGISLEQSYEDPKVACWLLDPDSQEPTLHSIVTSFLPHELPLLEGMETSQGIQS     2031
zPolθ PD    KILLLACELAVRGTFEDPKIACWLLDSSSKERTLHNMVTSFATEDLPMLEGISAGQGVQS     2033
            *****:* :::. ::****:****** .*:* ***.:****  .:**:***:.:.**:**

hPolθ PD    LGLNAGSEHSGRYRASVESILIFNSMNQLNSLLQKENLQDVFRKVEMPSQYCLALLELNG     2091
zPolθ PD    LGIYGEASQPGRYRAAIESVLVFRVMTQLNCLLEKDGFLDVFKKVEMPTQYCLALLELNG     2093
            **: . :.: *****::**:*:*. *.***.**:*:.: ***:*****:***********

hPolθ PD    IGFSTAECESQKHIMQAKLDAIETQAYQLAGHSFSFTSSDDIAEVLFLELKLPPNREMKN     2151
zPolθ PD    IGFSIAECEAQKHVMQAKLSALESQAYQLAGHSFSLTSPEDVAEVLFLELKLPPNGDLNG     2153
            **** ****:***:*****.*:*:***********:** :*:************* :::.

hPolθ PD    QGSKKTLGSTRRGIDNGRKLRLGRQFSTSKDVLNKLKALHPLPGLILEWRRITNAITKVV     2211
zPolθ PD    LKNKKTLGYTRR---AGARIKLSKQFSTTKDVLEKLKPLHPLPGVILEWRRITNALTKVV     2210
              .***** ***     *  :::*.:****:****:*** ******:**********:****

hPolθ PD    FPLQREKCLNPFLGMERIYPVSQSHTATGRITFTEPNIQNVPRDFEIKMPTLVGESPPSQ     2271
zPolθ PD    FPLQREKKWHSHLKMDRIHPISQSHTATGRVSFTEPNIQNVPKDFEIQMPTLIEESQTSQ     2270
            *******   : .* *:**:*:*********::**********:****:****: **  **

hPolθ PD    AVGKGLLPMGRGKYKKGFSVNPRCQAQMEERAADRGMPFSISMRHAFVPFPGGSILAADY     2331
zPolθ PD    NGGSKMWCKR-TKINR--LL--APLLKVSDKSPDKGMQFSVSMRHAFVPFSGGLILAVDY     2325
              *. :       * ::    :       ::.::: *:** **:********* ** ***.**

hPolθ PD    SQLELRILAHLSHDRRLIQVLNTGADVFRSIAAEWKMIEPESVGDDLRQQAKQICYGIIY     2391
zPolθ PD    SQLELRILAHLSRDRRLLHVLNSGADVFKSIAAEWKMVDPASVDDNMRQQAKQICYGIIY     2385
            ************:****::***:*****:********::* **.*::*************

hPolθ PD    GMGAKSLGEQMGIKENDAACYIDSFKSRYTGINQFMTETVKNCKRDGFVQTILGRRRYLP     2451
zPolθ PD    GMGAKSLGEQMGIEENDAACYIETFKSRYNGIQNFLRETVQKCGKNGYVKTLLGRKRFLP     2445
            *************:********::*****.**::*: ***::* ::*:*:*:***:*:**

hPolθ PD    GIKDNNPYRKAHAERQAINTIVQGSAADIVKIATVNIQKQLETFHSTFK-SHGHREGMLQ     2510
zPolθ PD    GIKDSNVYIKSHAERQAVNTTVQGSAADIVKLATINIQRRIEEAFPGVPTSHQHP-----     2500
            ****.* * *:******:** **********:**:***:::*  .  .  ** *

hPolθ PD    SDRTGLSRKRKLQGMFCPIRGGFFILQLHDELLYEVAEEDVVQVAQIVKNEMESAVKLSV     2570
zPolθ PD    ----SIRLGGRHRNQFRPLRGGYFILQLHDELIYEVAEEDVIQVAQIVKREMESVVKLYV     2556
                .:    : :. * *:***:*********:********:*******.****.*** *

hPolθ PD    KLKVKVKIGASWGELKDFDV   2590
zPolθ PD    KLRVKVKVGPSWGNLQDLDI   2576
            **:****:* ***:*:*:*:
```

**S1 Fig.**
