# Supplementary figures and images for: Zebrafish Polymerase Theta and human Polymerase Theta: Orthologues with homologous function
Source: PLoS One. 2025 Apr 29;20(4):e0321886. doi: 10.1371/journal.pone.0321886 (PMC12040184; doi:10.1371/journal.pone.0321886)

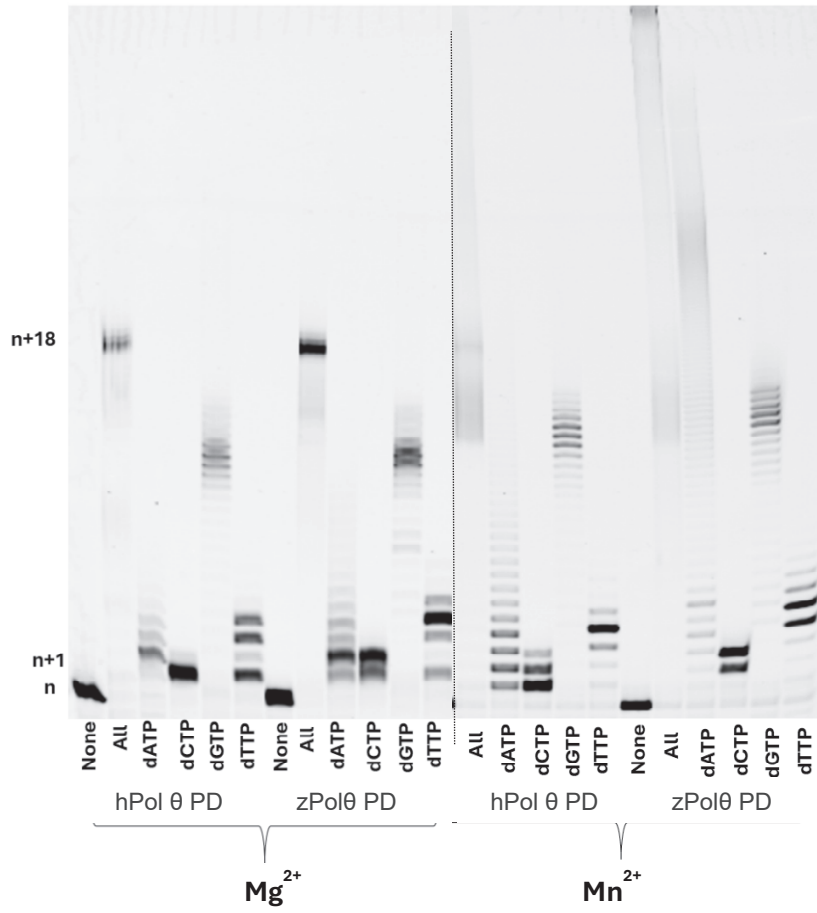

S2 Fig.

Supplement: S2 Fig — Both hPol θ PD and zPolθ PD were assayed under single-turnover conditions at t 4:1 ratio protein:DNA (see Materials and Methods). Pol θ and 25/40 dsDNA were preincubated and combined with either 10 mM MgCl2 or MnCl2 for 5 minutes and 37°C. DNA extension products were separated on a denaturing gel and visualized on a Typhoon scanner. (PDF) [file pone.0321886.s002.pdf]

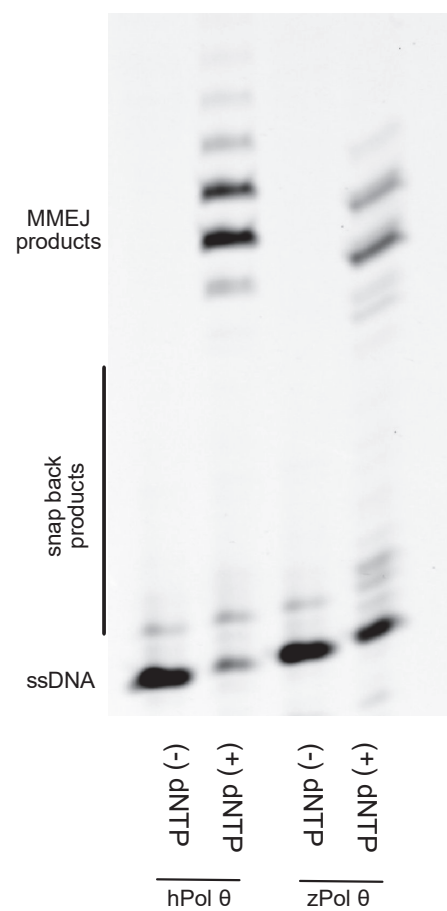

**S3 Fig.**

Supplement: S3 Fig — Samples either had all nucleotides (+ dNTP) or no nucleotides (-dNTP) added and the ternary complex was incubated for 45 minutes at 37°C. Reactions were stopped and products separated on a 12% Native PAGE. The gel was visualized on a Typhoon scanner. Start of MMEJ products are marked with an arrow, smaller snap-back products are indicated by the bracket. (PDF) [file pone.0321886.s003.pdf]

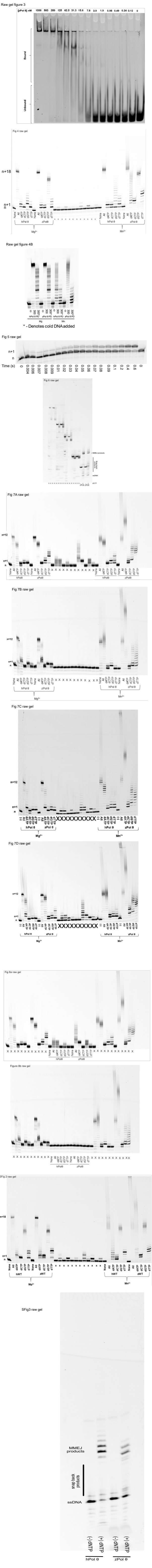

Supplement: S1 File — Raw gel images for the gels used in the figures as indicated by the label. “X” indicates a lane not used in the figure, labels correspond to labels in the figure. (PDF) [file pone.0321886.s004.pdf]
